# Training the osteoplastic flap technique in dogs

**DOI:** 10.1016/S1808-8694(15)31301-X

**Published:** 2015-10-20

**Authors:** Onivaldo Bretan, Emanuel Araújo Nogueira, Eriverton Ferreira da Silva, Sérgio Henrique K. Trindade

**Affiliations:** 1 Full Professor (Ph.D., Assistant Professor); 2 Resident physician, Discipline of Otorhinolaryngology; 3 Resident physician, Discipline of Otorhinolaryngology; 4 Resident physician, Discipline of Otorhinolaryngology

**Keywords:** osteoplastic flap, frontal sinus, technique

## Abstract

**Summary:**

Access to frontal sinus using the osteoplastic flap technique is indicated in lesions that do not yield endonasal approach. This technique can be practiced with dogs, although delineation of sinusal perimeter in canines is not as easy as it is in humans.

**Aim:**

This study aims at presenting a method to access and delineate the frontal sinus of canines to reproduce the osteoplastic flap technique in man.

**Study design:**

Surgical technique in animal.

**Material and Method:**

In adult dogs, two straight lines were drawn: one along the median line of the frontal region; and the other at 45° from the pupil. At the intersection point, 1-1.5 cm forward and 1 cm backward was measured; from these points, an incomplete rectangle delineating the frontal sinusal perimeters was drawn.

**Results:**

This procedure was performed 12 times during one year with participation of medical residents. Opening the animals' frontal sinuses was an easy procedure, and the osteoplastic technique to locate the sinus was performed without failures.

**Conclusion:**

The method of locating and delineating the frontal sinus of dogs was useful to show that the osteoplastic technique is realistically reproducible in men.

## INTRODUCTION

The osteoplastic technique to access the frontal sinus is indicated to treat lesions like osteomas and other neoplasias, cysts, mucoceles, frontal traumas, liquor fistulas, malignant neoplasias and cases of frontal sinusitis, when the endoscopic route is not indicated or accessible^1^, ^2^, ^3^, ^4^, ^5^, ^6^. Osteoplasty is relatively simple and can be performed with enlarged visualization of the surgical field^1^, ^3^. However, a few requirements should be fulfilled to perform a secure osteoplastic flap on the anterior wall of the frontal sinus. For those initiating in Otolaryngology, there are only a few opportunities to learn the technique due to a reduced number of cases indicated for this type of procedure^4^, ^5^, ^6^.

Due to great similarity of the correspondent region in men^7^, ^8^, ^9^, ^10^, ^11^, ^12^, ^13^, the use of dog's frontal sinus is a way to train this technique, which has been used for research trials in the last decades. However, there are some anatomical differences between the species: the canine frontal sinus is divided or composed by two or three compartments with distinct drainages and no communication among them: lateral, medial and rostral sinuses, the latter not recognized by some authors^14^, ^15^, ^16^. The lateral sinus is the larger, which is very long in dolichocephalic dogs and occupying great part of the frontal bone from the median line. The medial sinus may not be present in brachicephalic animals^14^, ^15^. The size and shape depend on skull format, the dog and the side^14^, ^15^, ^16^. The nasofrontal opening that links the lateral sinus is found in its medial region next to the intersinusal septum, just like in men^16^. Localization of the frontal sinus perimeters to perform osteoplasty in men requires Caldwell radiography^3^, which is not easily obtained in dogs. If the anatomical features of a dog's frontal sinus are similar to men, an adequate reproduction of this technique is possible, should the frontal sinus anatomy of the dog be well known. Perhaps because some authors' had different research purposes and gross localization of canine frontal sinus is relatively easy, they had not described the method used to operate animal models^7^, ^8^, ^9^, ^10^, ^11^, ^12^, ^13^. Bretan et al. (1983) reported a method that provided the delimitation of dogs' sinusal lumen. However, this method was useful just to remove the anterior wall of the frontal sinus for intrasinusal procedures, in which peculiar features of the osteoplastic technique were not considered. In addition, delineation of the sinusal cavity includes several steps, which are poorly and not clearly described by this study^17^. No reports were found in the literature presenting a method to approach the frontal sinus of a dog that allows training the technique to be reproduced in men. The objective of this study is to describe a practical method that localizes and delineates the frontal sinus of a dog, serving as a training practice of the osteoplastic technique's main steps for osteoperiostal flap of the anterior sinusal wall.

## MATERIAL AND METHODS

Adult dogs weighing 15-20 kg were placed in horizontal ventral decubitus position under Nembutal* anesthesia diluted with 30% intravenous sterile solution at Claude-Bernard position so that the frontal region was viewed horizontally. The frontal sinus was always accessed unilaterally. Physicians of the Residence Program of Otolaryngology, Technique and Experimental Surgery Laboratory, Departments of Ophthalmology, Otolaryngology and Head and Neck Surgery helped with these procedures, which were submitted for approval of the Ethics Commission on Animal Trials (protocol 401/2004).

## METHOD FOR FRONTAL SINUS IDENTIFICATION AND FLAP TECHNIQUE

**Figure 1:**
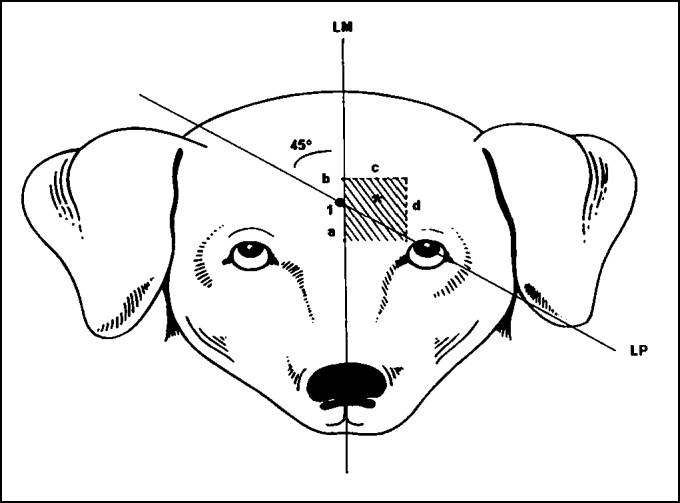
Lines and measures in the frontal region - illustration
LM = median lineLP = line along 45º of the pupil1 – intersection point of LM and LP linesa = 1-1.5cm backwardsb = 1-1.5 cm upwardsc = 1-1.5cm horizontally from LM lined = parallel line and A-B lines* = bold area indicates the assumed space of the frontal sinus

The method described was based on a previous trial, for which 28 animals were used^17^. Figure 1 illustrates the description as follows:

1. After antisepsis, an incision is done along the median line from the front facial region up to the initial portion of the dog's muzzle. This incision corresponds to a line defined as LM and may be deep, though without reaching the frontal bone or the periosteum. A subcutaneous dissection is performed to loose a broad superficial region and access the periosteum, which is rapidly reached as it is consisted of clear planes of easy segmentation (Figure 2). After reaching a comprehensive view of the frontal region, two repairing threads are used on the left and right of the incision borders, yielding a broad view of the field.

**Figure 2:**
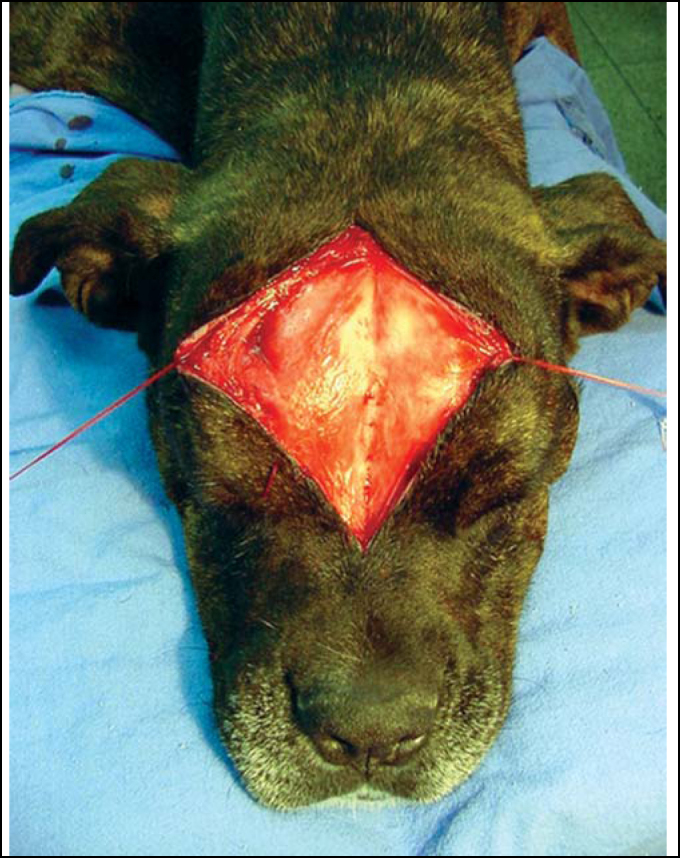
Exposure of the frontal periosteum
2. A second 45° line (LI) is drawn from the pupil up to the LM line; the meeting point of those lines corresponds to point 1. Both lines may be just mentally drawn or done with blue ink (methylene blue).
3. From point 1, depending on the dimensions of the frontal region, 1-1.5 cm backward and 1.5 cm upward is measured along the LM line (Figure 3). From the backward spot, 1.5-2.0 cm is measured lateral and horizontally and, then, parallel and upward in LM, delimiting an incomplete rectangle or a square, as shown on Figures 1 and 4. A horizontal line may be drawn, if preferred (Figure 3).

**Figure 3:**
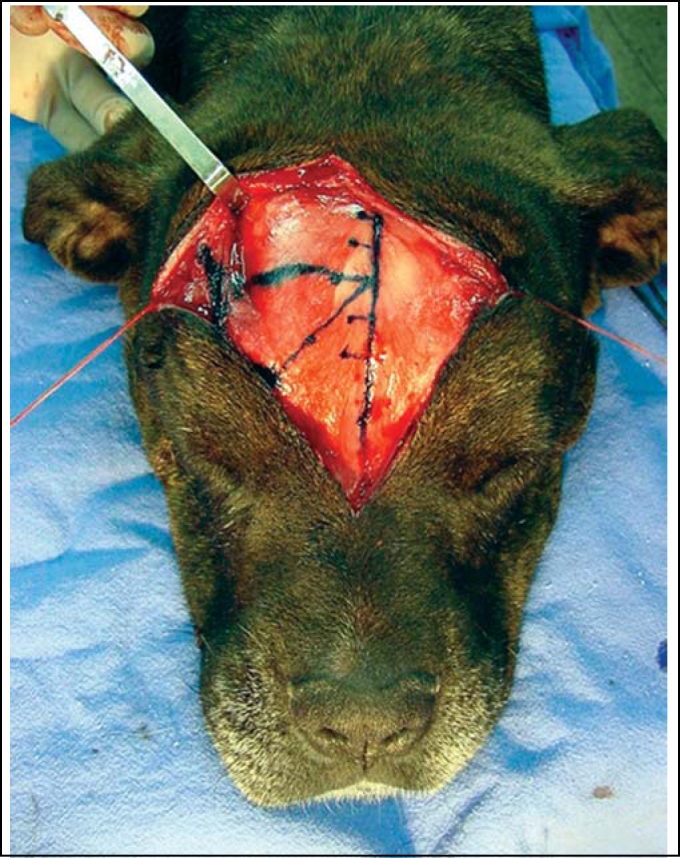
Delineating LM and LP straight lines and horizontal line (option).

**Figure 4:**
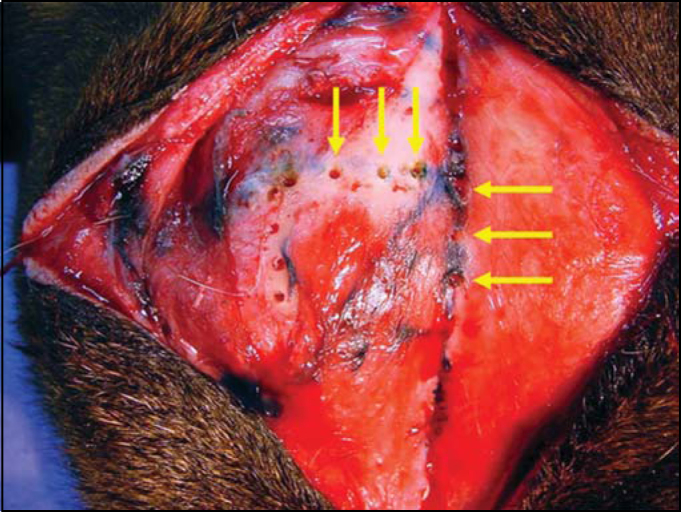
Arrows indicating holes at 45° curve to vertical line, with 3-4 mm of distance
4. The delimited rectangle corresponds, approximately, to a space planned on a previous model, which is supposed to be the left or the right sinus. After that, the following procedures are same as an osteoplasty in human^1^, ^3^. The holes done on the sinusal limits should use a fine drill, approximately at 45º curve to the subjacent sinus, resulting in an opening in wedge (Figure 5). The two maneuvers avoid great bone loss due to drill action, which would cause insertion of bone flap into the lumen, which should be fixed by steel thread. The distance between holes should not be greater than 3 to 4 mm. LM median line holes must also be at 45º curve, particularly to avoid the drill to meet the intersinusal septum. Laterally, the larger opening will eventually meet the insertion of the frontal muscle. Like in men, the periosteum should be separated at the hole line, cutting and dismissing it a few millimeters so that the drill does not get twisted. A fine scalpel should be used to link the holes to discharge the bone flap. A strong instrument, using some force but avoiding joint fracture, suspends this flap, which remains fixed to the circumjacent bone by the periosteum (Figure 6). The nasofrontal duct is identified and catheterized with injection of sterile solution, which should be drained (Figure 7). The bone flap is repositioned in its original place and sutured in two planes: periosteal and superficial.

**Figure 5:**
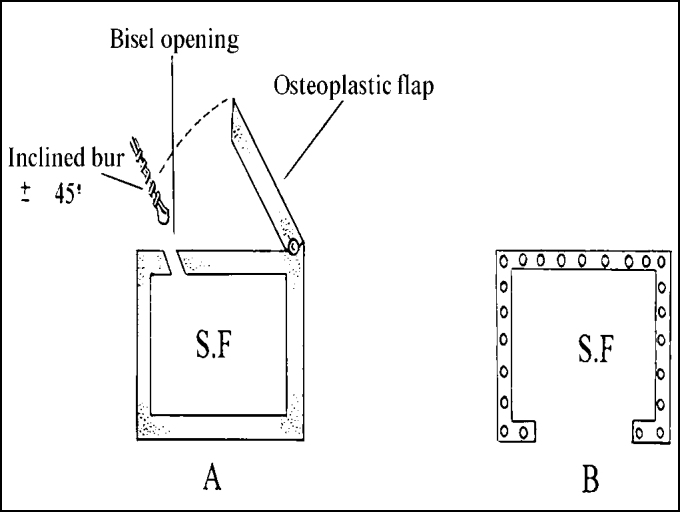
Illustration of the drill's position to make the holes and develop the osteoplastic flap with wedge borders.

**Figure 6:**
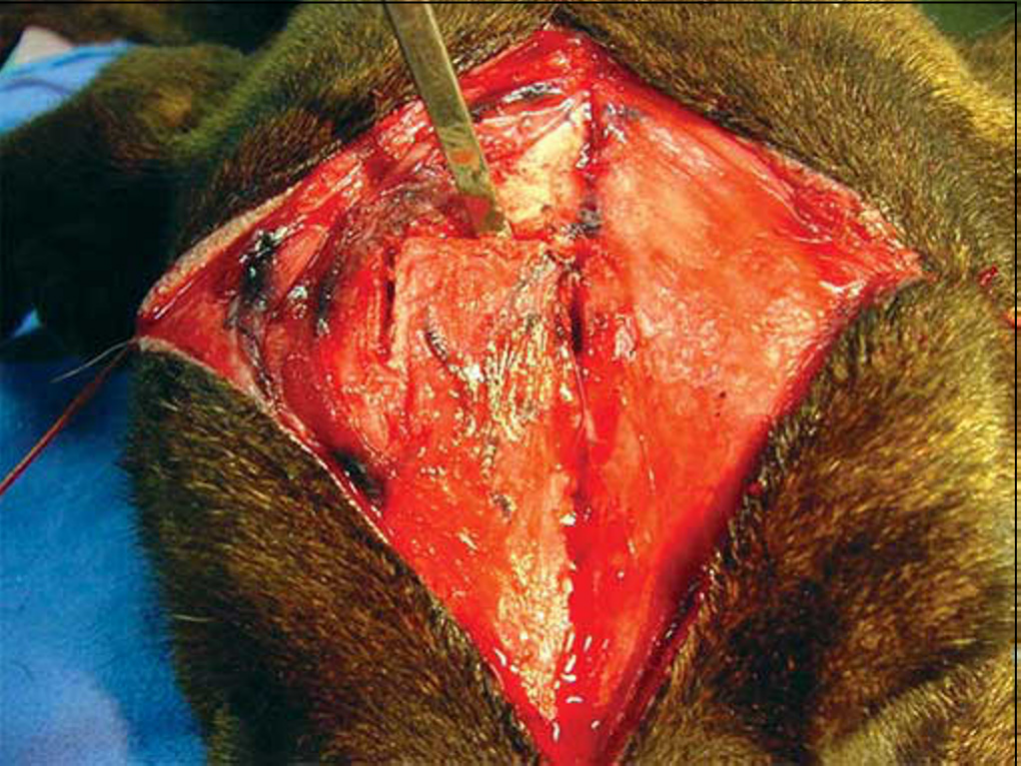
Sinus opening and flap elevation

**Figure 7:**
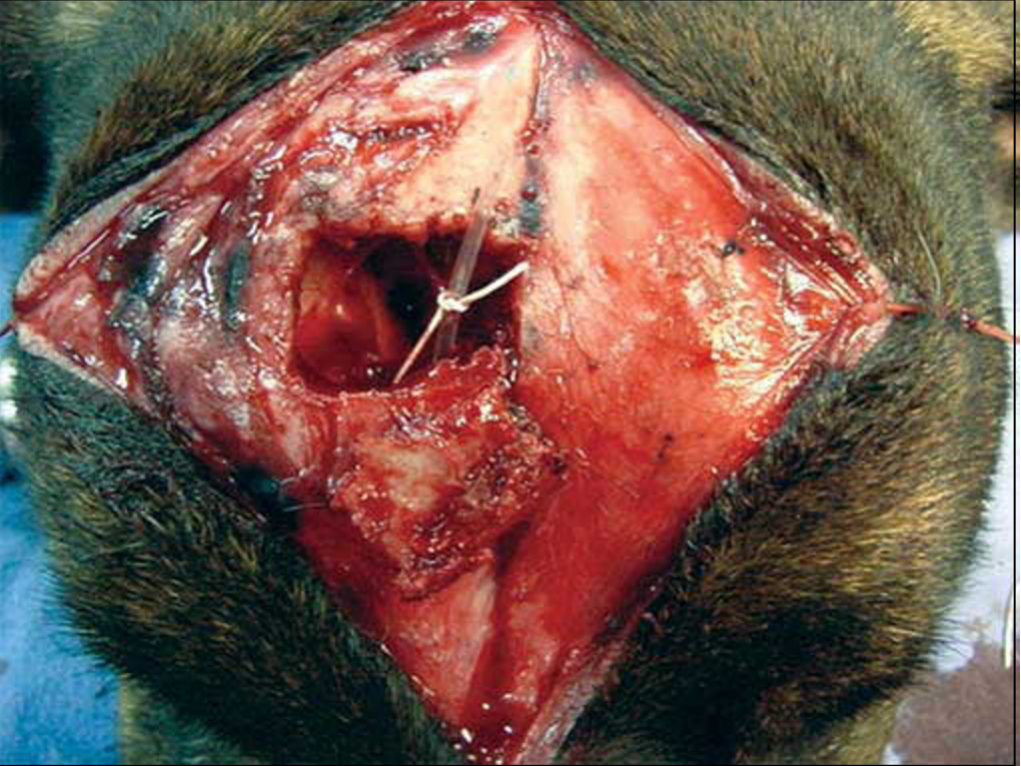
Osteoplastic flap fixed to the periosteum; insertion of catheter into the nasofrontal duct.

## RESULTS

In one-year period, 12 procedures were performed with participation of two medical residents. In all animals, sinus localization and delimitation was an easy and fast maneuver, whose method and technique were rapidly learned by the trainees. No failures have occurred in localizing or delimiting the sinusal cavity of all animals submitted to this procedure. Identification of three divisions of the sinus was not possible, so that the sinus found, according to anatomy books, was considered the lateral sinus.

The exposed sinus extended from the median line of the frontal bone going to its lateral and antero-posterior portion, revealing gross quadrangular shape. All animals showed medial nasofrontal communication near the intersinusal septum. In all dogs, a fine catheter was easily inserted in the nasofrontal lumen. Drainage of the solution injected was observed in the operated nostril of all animals. Grossly, the osteoplastic flap presented a rectangular shape with dimensions from approximately 2cm × 1.5cm to 2cm × 2.5cm.

## DISCUSSION

Surgical techniques used for distinct purposes are described in books and technical atlas. Montgomery reported the osteoplastic technique^4^ with details. Training these techniques in animal models is a way to maximize security and accuracy in performing them in humans, following the technical guides. The method presented in this study, a modification of a previous procedure, allowed a broader exposure of the dog's greater frontal sinus, most likely the lateral one, in all animals submitted to this approach. This procedure proved to be safe, that is, all perforations reached the sinus lumen, and was useful for the objective of this training, due to similarity of the frontal sinus position in men^14^. In all dogs, the opened cavity presented dimensions that allowed different procedures and practices for intrasinusal surgical acts. So far, even the small number of dogs was enough to verify that teaching the osteoplastic technique is feasible.

When practicing new techniques, it is always preferred to use live animals. However, similarly to dissections of ear and laryngeal structures, it is also possible to remove cephalic segments from animals used to other applications, which should be preserved in the refrigerator.

## CONCLUSIONS

Teaching the osteoplastic technique to reach a canine frontal sinus using the method of localization and delimitation of the sinusal space proved to be useful, once it can be reproduced in men.
